# Enhanced alcohol self-administration and reinstatement in a highly impulsive, inattentive recombinant inbred mouse strain

**DOI:** 10.3389/fnbeh.2013.00151

**Published:** 2013-10-30

**Authors:** Maarten Loos, Jorn Staal, August B. Smit, Taco J. De Vries, Sabine Spijker

**Affiliations:** ^1^Department of Molecular and Cellular Neurobiology, Center for Neurogenomics and Cognitive Research, Neuroscience Campus Amsterdam, VU University, AmsterdamNetherlands; ^2^Department of Anatomy and Neurosciences, Neuroscience Campus Amsterdam, VU University medical center, AmsterdamNetherlands

**Keywords:** BXD, inhibitory control, intra-individual variability, executive function, reinstatement, ethanol, 5csrt, addiction

## Abstract

Deficits in executive control have frequently been associated with alcohol use disorder. Here we investigated to what extent pre-existing genetically encoded levels of impulsive/inattentive behavior associate with motivation to take alcohol and vulnerability to cue-induced reinstatement of alcohol seeking in an operant self-administration paradigm. We took advantage of BXD16, a recombinant inbred strain previously shown to have enhanced impulsivity and poor attentional control. We compared BXD16 with C57BL/6J mice in a simple choice reaction time task (SCRTT) and confirmed its impulsive/inattentive phenotype. BXD16 mice were less active in a novel open field (OF), and were equally active in an automated home cage environment, showing that increased impulsive responding of BXD16 mice could not be explained by enhanced general activity compared to C57BL/6J mice. After training in a sucrose/alcohol fading self-administration procedure, BXD16 showed increased motivation to earn 10% alcohol solution, both under fixed ratio (FR1) and progressive ratio (PR2) schedules of reinforcement. Responding on the active lever readily decreased during extinction training with no apparent differences between strains. However, upon re-exposure to alcohol-associated cues, alcohol seeking was reinstated to a larger extent in BXD16 than in C57BL/6J mice. Although further studies are needed to determine whether impulsivity/inattention and alcohol seeking depend on common or separate genetic loci, these data show that in mice enhanced impulsivity coincides with increased motivation to take alcohol, as well as relapse vulnerability.

## Introduction

Deficits in executive functions, such as enhanced impulsivity and poor attentional control, have been associated with substance use disorders (Bickel et al., 2012), including that of alcohol (alcohol use disorder) (Verdejo-Garcia et al., 2008). Evidence from human prospective studies suggests that pathological levels of impulsivity may not only be a consequence of alcohol use, but could pre-date the development of alcohol use disorder (Caspi et al., 1996; Dawes et al., 1997). In line with these observations in humans, increased levels of impulsive behavior have been observed in alcohol-naïve rats (Steinmetz et al., 2000; Wilhelm and Mitchell, 2008), as well as in certain mouse lines (Logue et al., 1998; Oberlin and Grahame, 2009; Gubner et al., 2010) that are genetically predisposed to show a high preference of alcohol consumption. However, whether genetically determined poor executive control coincides with vulnerability to crucial stages of alcohol abuse, such as motivation to take and seek alcohol, and reinstatement of alcohol seeking after extinction, has not been investigated.

When kept under highly controlled conditions, differences between inbred mouse strains result from additive genetic effects and gene-by-environment interactions (Crabbe et al., 1999) and are therefore instrumental in understanding the genetic architecture of behavior. For instance, the BXD panel of recombinant inbred strains of mice, derived from an intercross of C57BL/6J and DBA/2J (BXD strains; Peirce et al., 2004) has previously been used to identify quantitative trait loci underlying aspects of executive control. Among 51 strains tested, BXD16 appeared to be the poorest performing strain in terms of discrimination reversal learning, a task proposed to target inhibitory control mechanisms (Laughlin et al., 2011). We recently showed that the same BXD16 strain was among the three poorest performing strains in a panel of 41 other BXD strains with respect to attentional performance in a 5-choice serial reaction time task (5CSRTT). In this task mice are required to respond to a brief (1 s) light stimulus in one of five response apertures. BXD16 mice showed a high intra-individual variability in correct response latencies as well as low response accuracy (Loos et al., 2012), both indicative of reduced attentional performance.

Here, we first independently validated previous data on inhibitory control and attention performance of BXD16 by using the simple choice reaction time task (SCRTT). The SCRTT in mice was developed to specifically measure attention in terms of intra-individual differences in response latencies, following the observation of increased intra-individual variability in response latencies in patients with Attention-Deficit/Hyperactivity Disorder (ADHD) that are putative indices of brief lapses in attention (Sergeant and Van Der Meere, 1990; Leth-Steensen et al., 2000; Klein et al., 2006; Bidwell et al., 2007; Spencer et al., 2009). Similar to the previously developed Go/No-Go task (Loos et al., 2010), in the SCRTT mice are required to fulfill an unpredictable number of responses in a start-response aperture before a Go-stimulus in a Go-response in the adjacent aperture is switched on. This procedure forces mice to remain close to the Go-response aperture, resulting in short Go-response latencies (Go-RT) and accurate measurement of intra-individual variability in Go-RT (Loos et al., 2010). Moreover, in order to motivate mice to respond quickly, the duration of the Go-stimulus was titrated such that 30% of initiated trials resulted in an omission. In addition, premature responses into the Go-response aperture before onset of the stimulus are not rewarded and signaled by a time out, resembling premature responding in the 5CSRTT (Loos et al., 2010), thereby providing an index of inhibitory control (i.e., motor impulsivity).

Subsequently, we compared BXD16 with C57BL/6J mice in an operant sucrose-fading alcohol self-administration protocol to address whether genetic differences in executive control coincide with the motivation to take and seek alcohol, and to reinstate alcohol seeking triggered by alcohol-associated cues.

## Materials and Methods

### Animals

C57BL/6J and BXD16 mice were obtained from Jackson Laboratory and bred in the facility of the Neuro-Bsik consortium of the VU University Amsterdam. During experiments, mice were singly housed on sawdust in standard Makrolon type II cages enriched with cardboard nesting material. Experiments were carried out in accordance with the European Communities Council Directive of 24 November 1986 (86/609/EEC), and with approval of the local animal care and use committee of the VU University.

#### SCRTT

Operant chambers (MEDNPW-5M Med Associates Inc., St Albans, VT, USA) were placed in sound-attenuating ventilated cubicles, and were equipped with five response holes, and, at the opposite wall, a food magazine with a reward dispenser (14 mg Dustless Precision Pellets; Bio-Serve, Frenchtown, NJ, USA) and a house light. Both response holes and the food magazine contained yellow LED stimulus lights and infrared response detectors. Male 8 weeks old mice (C57BL/6J *n* = 10; BXD16 *n* = 9), kept on an individual feeding regime to maintain body weight at 90% of their free feeding weight, were trained (5 days each week, 30 min per session throughout the experiment) to earn food rewards by responding into one of five response holes in operant cages during the light phase, as described in detail previously (Loos et al., 2009, 2010). The cue light in the middle of the five response holes (i.e., hole three) was designated as start-stimulus, and the cue light in the response hole immediately to the left or right (i.e., hole 2 or 4, counterbalanced across strains) was designated as Go-stimulus. During the first four training sessions, a trial started with the illumination of the start-stimulus. A response into the start-stimulus hole extinguished the start-stimulus and switched on the Go-stimulus. A Go-response into the Go-stimulus hole switched off the stimulus and was immediately followed by distribution of a reward into the magazine. After an interval of 5 s, the next trial commenced. Premature start and Go-responses in non-illuminated response holes, as well as perseverative start-responses after presentation of the Go-stimulus were not rewarded but followed by a 5 s time-out (TO) period during which house light and stimulus light were switched off.

In subsequent sessions, responding at a variable ratio 3 scheduled into the illuminated start-stimulus hole was required to ignite the Go-stimulus. The Go-stimulus was only switched on for the duration of an individually-titrated limited hold (LH) period, which was set to 5 s during the first session. A Go-response during the LH period resulted in the delivery of a reward, whereas an omission of a Go-response was followed by a 5 s TO period. The percentage of omissions of Go-responses in each sessions was defined as: 100^*^ [omissions Go-response / (omissions Go-response + correct Go-responses + perseverative start-responses)]. To titrate the percentage of omissions to 30% for each subject in subsequent sessions, LH periods were decreased 0.7 fold if the percentage of omissions during the previous session was less than 25%, and increased by 1.25 fold if the percentage of omissions during the previous session was larger than 35%. Impulsivity in the SCRTT was defined as the percentage of premature Go-responses, calculated as: 100^*^ [number trials premature Go-response / number started trials]. Furthermore, we recorded the latencies between the onset of the Go-stimulus and a Go-response into the Go-stimulus hole (GoRT). Prior experiments indicated that GoRTs > 1.7 s are observed when mice travel to the magazine in between start and Go-responses. Therefore, GoRTs > 1.7 s were excluded in the calculation of mean GoRT, mode of GoRTs and variability of GoRTs (standard deviation of GoRTs; stdev). After a stable mean GoRT and percentage of premature responses in three consequent sessions for either strain, data of the subsequent sessions was taken as average SCRTT performance.

#### Activity/Anxiety test battery

As part of a larger screening project of BXD and common inbred lines, BXD16 and C57BL/6J mice were subjected to automated home cage testing of activity (unpublished; BXD16 *n* = 9, C57BL/6J *n* = 105) followed by a battery of conventional activity/anxiety tests (unpublished; BXD16 *n* = 12, C57BL/6J *n* = 52) as described previously (Loos et al., 2009). After arrival in the screening facility (7:00 lights on, 19:00 lights off) at the age of 7 weeks mice were individually housed and testing started 1 week later in the order described below. All mice were subjected to all behavioral tests, and the order of the tests was identical for all mice. All testing occurred during the light phase (between 8:00 and 12:00). On testing days, mice were transferred one by one from the housing room to the testing room and immediately introduced into the test apparatus.

##### Automated home cage activity.

Individual mice were housed in a home cage environment (PhenoTyper model 3000, Noldus Information Technology, Wageningen, The Netherlands) for seven consecutive days, as described in detail previously (Maroteaux et al., 2012). Mice were introduced in the cage in the second half of the subjective light phase (14:00 h–16:00 h), and video tracking started at the onset of the first subjective dark phase (19:00 h). The cages (*L* = 30 × *W* = 30 × *H* = 35 cm) were made of transparent Perspex walls with an opaque Perspex floor covered with bedding based on cellulose. A feeding station and a water bottle were attached onto two adjacent walls. A triangular shaped shelter compartment (height: 10 cm; non-transparent material) with two entrances was fixed in the corner of the opposite two walls. The top unit of each cage contained an array of infrared LEDs and an infrared-sensitive video camera used for video-tracking. The X-Y coordinates of the center of gravity of mice, sampled at a resolution of 15 coordinates per second were acquired and smoothed using EthoVision software (EthoVision HTP 2.1.2.0, based on EthoVision XT 4.1, Noldus Information Technology, Wageningen, The Netherlands) and processed to generate the total distance moved per 12 h time bins by AHCODA^TM^ (Synaptologics BV, Amsterdam, The Netherlands) as described previously (Maroteaux et al., 2012).

##### Novelty induced hypophagia

After testing in the automated home cage mice were transferred to standard individual housing conditions for at least 6 days. During this habituation phase a metal food cup was present in their cage, and mice were familiarized with an appetitive snack (cream cracker) placed in a metal food for three times. On two subsequent days the latency to start eating the snack was scored (maximum duration 240 s). If the subject did not start eating within 240 s, the maximum time was assigned. The mean home cage latency (HC latency) to eat was calculated over 2 days. On the next day, and 1 week later, mice were transferred to a novel clean cage with fresh bedding containing the metal cup with the familiar snack. Both the latency until the first touch of the cup and latency to start eating the snack were recorded manually. The mean novel home cage latency (NHC latency) of both days was used for analysis. If a subject did not eat within 720 s, the maximum time was assigned.

##### Novel object exploration

On two different days, novel objects (metal ring and a blue plastic bottle cap respectively) were introduced into the home cage. Both latency until first touch of the object and cumulative time touching the object during 4 min were recorded manually. If a subject did not touch the object during the test, the latency was set to 240 s. The mean of both novel object sessions was used for analysis.

##### Dark-light box (DLB)

Mice were introduced into the dark compartment (< 10 lx, length × width × height: 20 × 20 × 30 cm) of a DLB, and 60 s later the door opened providing access to an identical sized compartment which was brightly lit (625 lx) and left open for 10 min. Visits to, and time spent in the light compartment were counted when the body reference point of a mouse protruded at least 2 cm into the light compartment away from the door (12.5 frames/s, EthoVision 3.0, Noldus Information Technology).

##### Elevated plus maze (EPM)

Mice were introduced into the closed arm of an Elevated plus maze (EPM; arms 30 × 6 cm, walls 35 cm high, elevated 50 cm above the ground), facing the closed end of the arm. The EPM was illuminated with a single white fluorescent light bulb from above (130 lx) and exploratory behavior was video tracked for 5 min (12.5 frames/s, EthoVision 3.0, Noldus Information Technology). The border between center and arm entries was defined at 2 cm into each arm, producing the number of entries into the open arms, into the closed arms, onto the center platform, and time spent on the open arms. In addition, latency to explore was defined by the time between introduction onto the maze and the first appearance in the maze center.

##### Open field (OF)

Mice were introduced into a corner of the white square open field (OF; 50 × 50 cm, walls 35 cm high) illuminated with a single white fluorescent light bulb from above (130 lx), and exploration was tracked for 10 min (12.5 frames/s; EthoVision 3.0, Noldus Information Technology). Time spent in, and number of entries into the center square area (20 × 20 cm) was measured using EthoVision. The Strategy for the Exploration of Exploration software (SEE; Kafkafi et al., 2005) was used to smoothen path shape to calculate the total distance moved. Furthermore, SEE uses the distribution of speed peaks to parse the locomotor data into slow local movements (lingering episodes) and progression segments, which together constitute all distance traveled. In addition to the traditional measures in the OF, describing the animal tendency to engage in exploratory behavior, SEE was used to calculate the number of progression segments and the median duration of a lingering episode. SEE also enables the calculation of measures that describe the strategy of movement once exploration has been initiated: the median distance traveled per progression segment, the median duration of a progression segment, the number of stops per distance and the median acceleration during a progression.

#### Operant alcohol self-administration

A third group of male 10 weeks old BXD16 (*n* = 10) and C57BL/6J (*n* = 9) mice was trained in operant conditioning cages in sound attenuating chambers (TSE Systems, Bad Homburg, Germany) for 5 days per week (60 min per session throughout the experiment) during the subjective dark phase. Food and water were available *ad libitum* in the home cage. During training, a red house light mounted outside the operant box, but inside the chamber, provided dim illumination of the chamber. Two levers, of which one was active, were located at opposite sides of the cage. A predefined number of responses onto the active lever resulted in delivery of 10 µl liquid reward into the receptacle (left of active lever), and switched on a white stimulus light located above the receptacle for 2 s. After a TO period of 15 s, during which lever presses were without consequence but recorded (i.e., TO responses), a red cue light above the receptacle was switched on. The preference of responding onto the active lever (active lever preference) was defined as follows: [number active lever responses / (number active lever responses + number inactive lever responses)].

Mice were trained to respond for a 10% alcohol solution using a sucrose fading protocol at a fixed ratio 1 (FR1) schedule of reinforcement, in which reward consisted of the following solutions (wt/vol in tap water): 10% sucrose in session 1–9 (S1–S9), 10% sucrose and 2% alcohol (S10–S13), 10% sucrose and 4% alcohol (S14–S15), 10% sucrose and 6% alcohol (S16–S17), 10% sucrose and 8% alcohol (S18–S20), 10% sucrose 10% alcohol (S21–S25), 5% sucrose and 10% alcohol (S26–S31) and finally 10% alcohol (S32–S37). In the subsequent five sessions (S38–S42), mice were subjected to a progressive ratio (PR2) schedule. During the initial four PR2 sessions mice were habituated to the procedure and not analyzed, data of the 5th session was used to investigate motivation. In the 5th session a breakpoint was determined as the last completed ratio. Next, mice received six sessions (S43–S48) of FR1 training for 10% alcohol. During the subsequent 20 sessions (S49–S68) of extinction training, responding on the previously active lever was without programmed consequences. During the cue-induced reinstatement session (S69), reinstatement of alcohol seeking was determined in response to presentation of alcohol-conditioned (compound) cues: cue lights in response to active lever responses and a droplet of 10% alcohol (10 μl) placed in the receptacle at the start of the session to provide the olfactory cue (scent) and gustatory cue (taste) of alcohol. Behavior during the reinstatement session was compared with the last extinction session.

### Statistical analyses

For evaluation of strain differences, analysis of variance (ANOVA) was used with “strain” as between-subjects factor. Task manipulations across different sessions were analyzed using ANOVA with repeated measures with “session” as within-subjects factor. When Mauchly’s test for sphericity of data was significant, more conservative Huynh–Feldt corrected degrees of freedom and related probability values were reported. Where appropriate, post hoc tests were performed with Student’s *t*-tests for “strain” effects and paired Student’s *t*-tests for “session” effects. All data are depicted as means ± standard error of the mean (SEM), and the level of significance was set at *P* < 0.05.

## Results

### SCRTT

**Figure 1:**
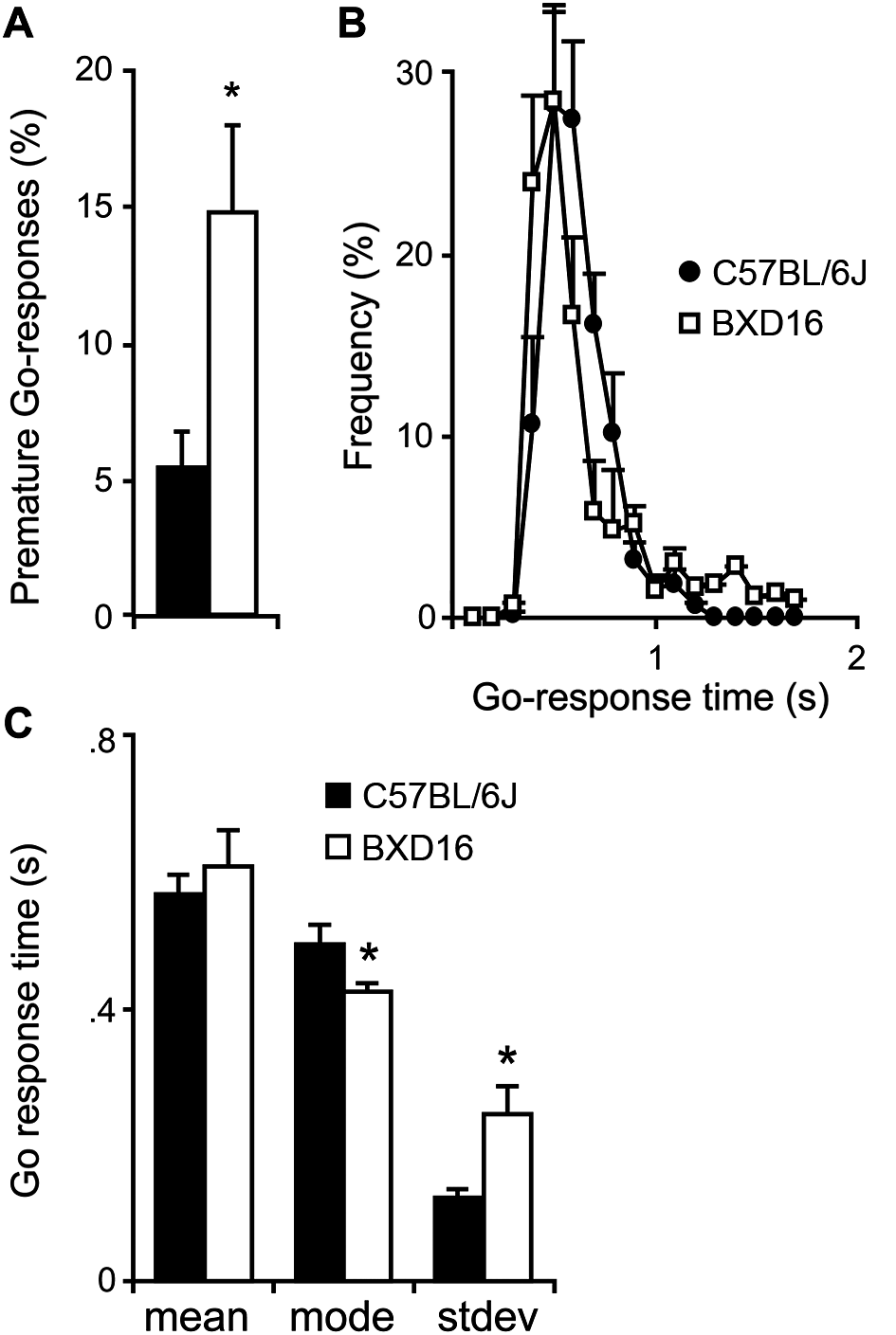
**Differences in impulsivity and attention between BXD16 and C57BL/6J in the SCRTT**. **(A)** BXD16 mice were more impulsive in terms of number of premature Go-responses. **(B)** Graphical representation and **(C)** quantification of distribution of Go-responses, indicating higher response variability (standard deviation: stdev) in BXD16 than C57BL/6J, indicative of more frequent lapses in attention. BXD16 mice have a lower mode of Go-responses than C57BL/6J. *P < 0.05.

BXD16 mice were more impulsive in terms of percentage of premature Go-responses during presentation of the start stimulus compared with C57BL/6J mice (Figure 1A; strain: *F*(1,17) = 6.87, *P* < 0.05). Although the required LH time was significantly longer for BXD16 mice (strain: *F*(1,17) = 8.85, *P* < 0.01), their mean GoRTs were not different from C57BL/6J mice (Figure 1B; strain: *F*(1,16) = 0.48, ns). This was explained by the observation that BXD16 mice had a faster mode of GoRTs (Figure 1C; strain: *F*(1,16) = 4.72, *P* < 0.05), but also a larger GoRT variability (Figure 1C, stdev; strain: *F*(1,16) = 8.73, *P* < 0.01). No significant differences were found in the number of initiated trials (strain: *F*(1,17) = 3.40, ns) and the percentage of omission of Go-responses (strain: *F*(1,17) = 3.87, ns). Thus, in the SCRTT we observed enhanced impulsivity and reduced attentional performance in BXD16 mice, in line with previous studies (Laughlin et al., 2011; Loos et al., 2012).

### Battery of activity/anxiety tests

**Figure 2:**
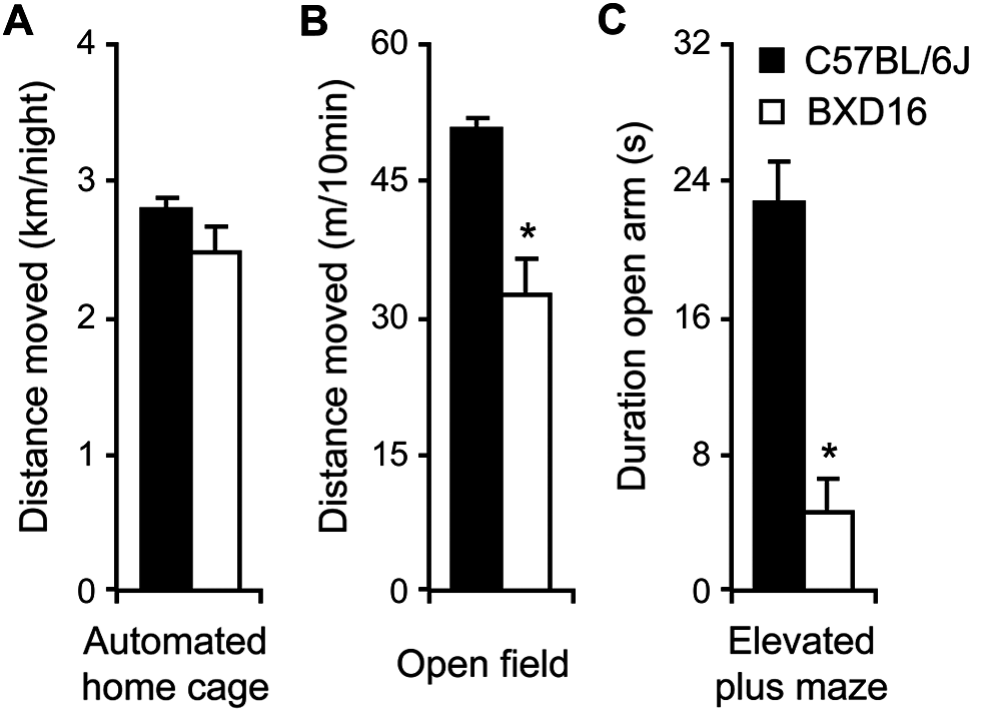
**Comparison activity/anxiety of C57BL/6J and BXD16**. **(A)** Total distance moved during the 12 h of the third dark phase in the automated home cage. **(B)** Total distance moved during a 10 min novel OF exposure. **(C)** Time spent on the open arm during a 5 min elevated plus maze exposure.

With respect to activity, during the 3rd day in the automated home cage environment, i.e., after the effect of novelty on activity levels during the 1st days had largely faded (De Visser et al., 2006), activity of BXD16 was not different from C57BL/6J in terms of total distance moved during the dark phase (Figure 2A; *F*(1,112) = 1.39, ns) or during the light phase (Table 1). Moreover, during a 10 min novel OF session, BXD16 were significantly less active compared with C57BL/6J mice (Figure 2B; *F*(1,62) = 28.00, *P* < 0.001). In addition, several other activity related measures, such as the number of entries into the closed arm of an EPM, showed a reduction of activity in BXD16 compared with C57BL/6J mice (Table 1). BXD16 displayed more anxiety in terms of a reduction of the time spent on the open arms of the EPM (Figure 2C; *F*(1,62) = 13.52, *P* < 0.001) and in the light compartment of the DLB (Table 1) compared with C57BL/6J mice. Taken together, the increase in impulsive responding of BXD16 mice in the SCRTT could not be explained by higher levels of general activity.

**Table 1:** **Activity and anxiety-related behavior in C57BL/6J and BXD16**.

		**C57BL/6J**	**BXD16**
**Automated home cage**			
Total distance during dark phase	m	280.51+/−8.02	247.46+/−20.43
Total distance during light phase	m	40.66+/−2.02	36.21+/−9.87
**Novelty induced hypophagia**			
Latency to eat in home cage	sec	31.83+/−4.86	66.29+/−18.13*
Latency to eat in novel home cage	sec	150.84+/−15.80	143.54+/−62.09
**Novel object exploration**			
Latency to explore novel object	sec	47.97+/−6.41	53.26+/−22.18
Duration exploration novel object	sec	20.45+/−1.88	25.22+/−4.00
**Dark Light box**			
Duration in the light	sec	344.04+/−6.01	292.28+/−20.37**
Entries into the light	number	44.33+/−1.29	35.00+/−3.01**
**Elevated plus maze**			
Duration on the open arms	sec	22.85+/−2.33	4.61+/−1.91***
Entries into open arms	number	5.21+/−0.4	1.92+/−0.60***
Entries into center	number	28.29+/−0.92	14.33+/−1.94***
Entries into closed arms	number	23.71+/−0.8	13.08+/−1.64***
Latency to enter the center	sec	9.53+/−0.96	28.81+/−15.02*
**Open Field**			
Total distance traveled	cm	5081.78+/−141.59	3272.30+/−364.36***
Duration in center	sec	68.87+/−3.5	60.52+/−14.94
Entries into center	number	44.17+/−2.08	30.67+/−5.72*
Number of progressions	number	152.79+/−4.33	100.25+/−13.20***
Distance traveled per progression	cm	25.52+/−0.68	24.00+/−3.18
Duration of a lingering episode	sec	1.19+/−0.05	1.97+/−0.32***
Duration of a progression	sec	1.54+/−0.03	1.67+/−0.16
Acceleration	cm/sec2	16.12+/−0.25	12.93+/−0.62***
Number of stops per distance	number/cm	0.03+/−0.00	0.03+/−0.01

*P < 0.05, **P < 0.01, ***P < 0.001.

### Alcohol self-administration

For each training phase in the sucrose-to-alcohol fading self-administration paradigm, we measured the number of earned rewards and the preference for the active lever (primary outcome measure of reward seeking), the number of non-rewarded active lever responses during the TO responses (a measure associated with an impulsive/compulsive phenotype; Deroche-Gamonet et al., 2004; Ghitza et al., 2006; Diergaarde et al., 2009) and inactive lever responses (measure of general activity).

#### 

##### Acquisition of lever pressing under sucrose reinforcement (S1–S9)

**Figure 3:**
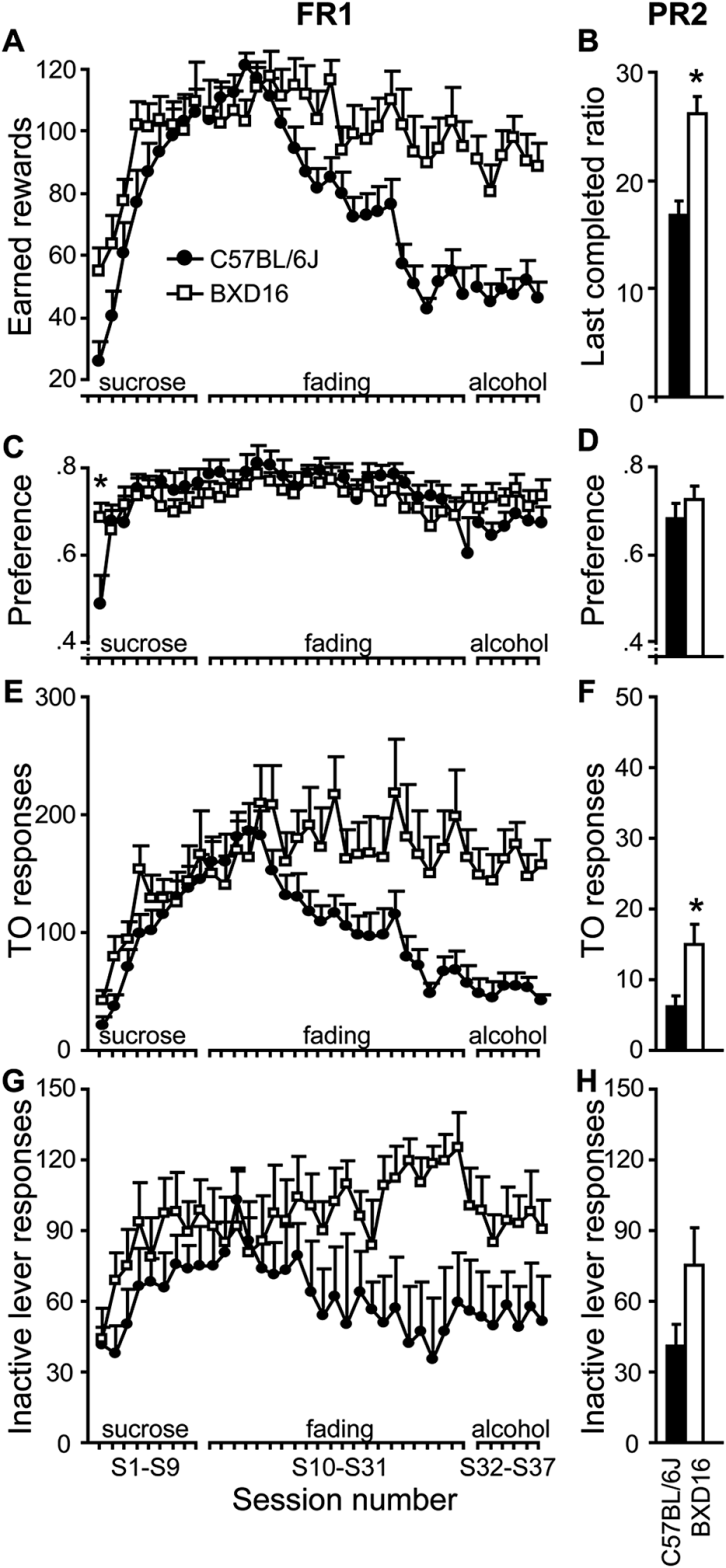
**Enhanced motivation for alcohol in BXD16 mice**. Under an FR1-schedule of reinforcement, **(A)** BXD16 mice earned a significant higher number of rewards when alcohol was added to the reward (S10-S31), which persisted until mice responded for a 10% alcohol solution (S32–S37). **(B)** Under a PR2 schedule of responding for 10% alcohol reward, BXD16 mice reached a higher last completed ratio than C57BL/6J. **(C,D)** BXD16 mice showed a faster development of dissociation of active and inactive lever compared with C57BL/6J and showed equal preference during FR1 and PR2 responding for 10% alcohol. **(E,F)** BXD16 showed enhanced TO responding on the active lever during FR1 and PR2 responding for 10% alcohol compared with C57BL6J. **(G,H)** BXD16 and C57BL/6J mice did not show a significant difference in inactive lever responding during FR1 and PR2 responding for 10%. For statistical evaluation of results, see Table 2 and text. *P < 0.05.

During the first acquisition sessions, active lever responding was rewarded with 10% sucrose solution droplets (for statistics see Table 2). The number of earned rewards increased readily across sessions (Figure 3A) without significant differences between strains. The preference for the active over the inactive lever also increased in both strains (Figure 3C), and this increase developed faster in BXD16 compared with C57BL/6J mice. Total number of TO responses (Figure 3E) and inactive responses (Figure 3G) increased significantly across acquisition sessions, but there was no strain difference. Together, these data indicate a faster development of the dissociation between active versus inactive lever in BXD16 mice compared with C57Bl/6J mice, and the absence of strain differences in responding for 10% sucrose reward.

**Table 2:** **Statistical data of sucrose to alcohol fading (FR1)**.

	**Sucrose acquisition**	**Sucrose to alcohol fading**	**FR1 alcohol**
**Earned rewards**			
Session	*F*(5.45,92.63) = 40.80***	*F*(21,357) = 20.06***	*F*(5,85) = 2.25
Strain	*F*(1,17) = 1.84	*F*(1,17) = 5.51*	*F*(1,17) = 18.78***
Session*Strain	*F*(5.45,92.63) = 2.20	*F*(21,357) = 8.50***	*F*(5,85) = 1.34
**Preference for active lever**			
Session	*F*(5.58,94.86) = 5.89***	*F*(21,357) = 3.59**	*F*(2.27,38.57) = 0.64
Strain	*F*(1,17) = 0.001	*F*(1,17) = 0.41	*F*(1,17) = 2.53
Session*Strain	*F*(5.58,94.86) = 3.54**	*F*(21,357) = 1.24	*F*(2.27,38.57) = 0.65
**Active lever responses during TO**			
Session	*F*(4.44,75.54) = 14.79***	*F*(21,357) = 4.21***	*F*(3.73,63.46) = 1.37
Strain	*F*(1,17) = 1.38	*F*(1,17) = 4.2	*F*(1,17) = 21.51***
Session*Strain	*F*(4.44,75.54) = 0.73	*F*(21,357) = 3.61***	*F*(5,85) = 0.44
**Inactive lever responses**			
Session	*F*(8,136) = 11.04***	*F*(21,357) = 0.54	*F*(5,85) = 0.72
Strain	*F*(1,17) = 1.45	*F*(1,17) = 3.46	*F*(1,17) = 3.58
Session*Strain	*F*(8,136) = 1.05	*F*(21,357) = 2.58***	*F*(5,85) = 0.20

*P < 0.05, **P < 0.01, ***P < 0.001.

##### Sucrose to alcohol fading (S10–S31)

Across 22 training sessions, alcohol was added to the rewarding solution up to a concentration of 10%, and sucrose was subsequently faded out (for statistics see Table 2). BXD16 and C57BL/6J mice reacted differently to the change of rewarding solution in terms of number of earned rewards, with BXD16 mice earning more rewards from session 20 onwards (10% sucrose, 8% alcohol) than C57BL/6J mice (Figure 3A). During this training phase, the preference for the active lever remained, without any strain difference (Figure 3C). BXD16 and C57BL/6J mice reacted differently in terms of the number of active responses during the TO, which started to differ at session 24 (10% sucrose with 10% alcohol; Figure 3E). There was no overall strain difference in inactive lever responding (Figure 3G), although there was a significant session^*^ strain effect. Together these data show that C57BL/6J earned less rewards than BXD16 mice when alcohol was added to the rewarding solution, and that BXD16 showed more TO responding, possibly related to an impulsive/compulsive phenotype (Deroche-Gamonet et al., 2004; Ghitza et al., 2006; Diergaarde et al., 2009).

##### FR1 responding for a 10% alcohol reward (S32–S37)

Next, mice responded at an FR1 schedule for a 10% alcohol solution (for statistics see Table 2). BXD16 mice earned substantially more rewards than C57BL/6J mice (Figure 3A). Nonetheless, the preference for the active lever remained high in both strains and did not differ between strains (Figure 3C), indicating that the 10% alcohol solution was also reinforcing in C57BL/6J mice. Again, BXD16 mice made significantly more TO responses (Figure 3E). There was no significant difference in inactive lever responding between strains (Figure 3G). Together these FR1 data suggest that, in addition to an impulsive phenotype, BXD16 mice showed an enhanced motivation to self-administer alcohol, which was further investigated in a PR2 schedule of reinforcement.

##### Motivation for alcohol under a PR2 schedule of reinforcement (S38–S42)

To assess motivation of mice to earn a 10% alcohol reward, the response requirement was increased progressively with two responses after each reward (i.e., a PR2 schedule of reinforcement). Mice were acquainted to the PR2 schedule for four sessions and motivation to earn alcohol was assessed during the 5th PR2 session. BXD16 mice reached a higher breakpoint (i.e., last completed ratio) than C57BL/6J mice (Figure 3B; strain: *F*(1,17) = 20.01, *P* < 0.001). No difference was detected in the preference for the active versus inactive lever (Figure 3D; strain: *F*(1,17) = 0.71, ns). Again, BXD16 mice made significantly more TO responses (Figure 3F; *F*(1,17) = 7.67, *P* < 0.05). There was no strain difference in inactive lever responding (Figure 3H; *F*(1,17) = 2.84, ns). In conclusion, BXD16 mice appeared to be more motivated to respond for alcohol reward than C57BL/6J mice (PR).

##### Extinction rate of alcohol seeking

**Figure 4:**
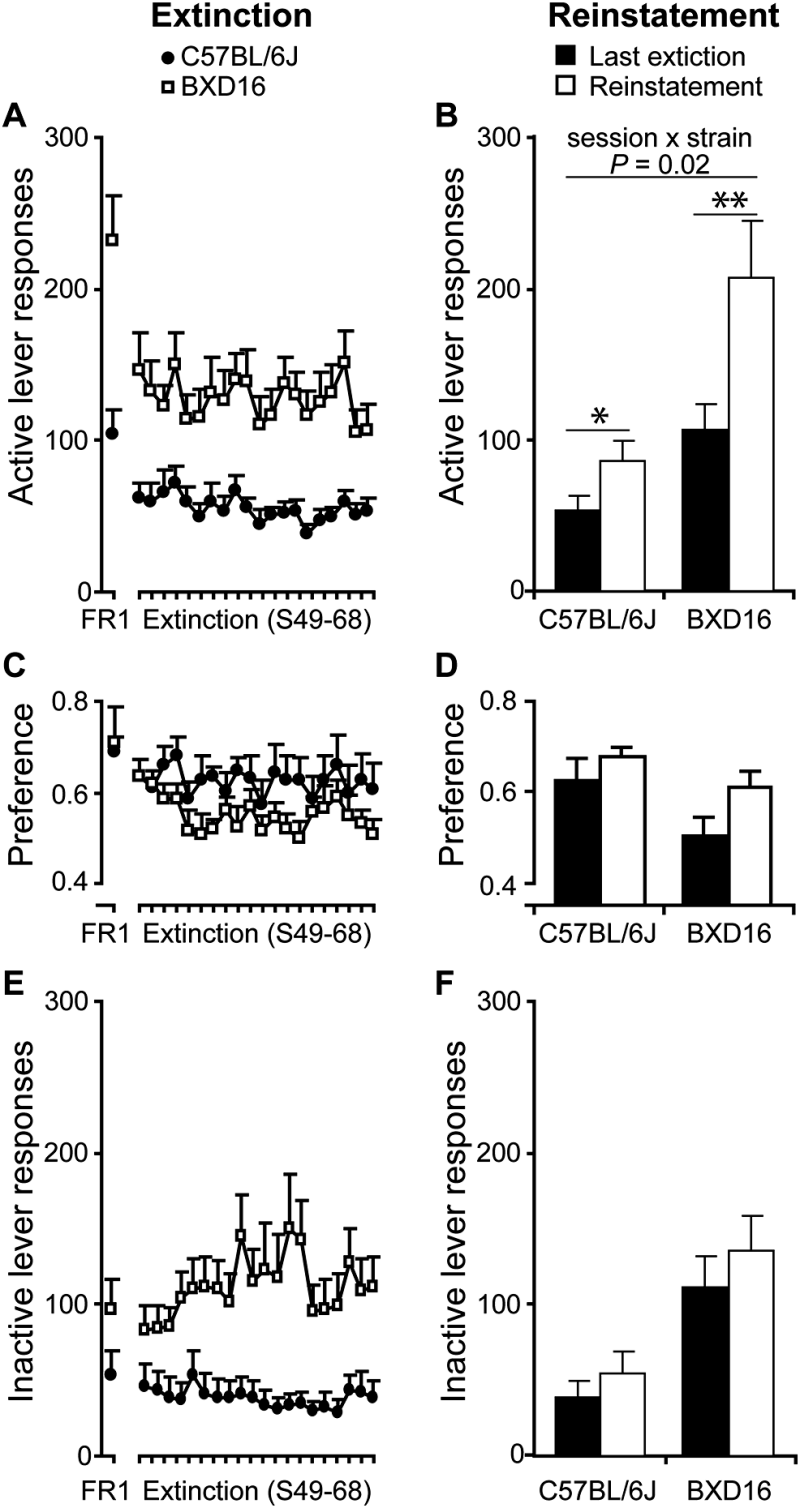
**Stronger reinstatement of alcohol seeking in BXD16 mice**. **(A)** Responding at the previously active lever decreased equally in both strains compared to the last FR1 session during extinction session, and **(B)** increased during the reinstatement session, with a stronger increase in BXD16 compared with C57BL/6J mice. **(C)** The preference for the active lever decreased during extinction compared with the last FR1 session, with no differences between strains and **(D)** increased during the reinstatement session compared with the last extinction session with no difference in this increase between strains. **(E)** Inactive lever responding did not decrease during extinction in comparison with the last FR1 session, but **(F)** significantly increase during the reinstatement session with no differences in this increase between strains. *P < 0.05, **P < 0.01.

Over the course of the last FR1 session (S48) and 20 subsequent extinction sessions (S49–S68), alcohol seeking behavior in terms of number of responses on the previously active lever decreased (Figure 4A; session: *F*(20,320) = 8.57, *P* < 0.001). Post-hoc testing indicated that this decrease was significant immediately during the first extinction session compared with the last FR1 session (*P* < 0.01). No difference in the rate of active lever extinction was detected between strains (session × strain: *F*(20,320) = 1.54, ns), but there was a significant higher rate of active lever responding in BXD16 mice (strain: *F*(1,16) = 17.3, *P* < 0.001). There was a significant decrease in the preference of the active over the inactive lever in both strains (Figure 4C; session: *F*(20,320) = 3.36, *P* < 0.001; session × strain: *F*(20,320) = 1.06, ns) without a difference between strains (strain: *F*(1,16) = 1.64, ns). The number of responses on the previously inactive lever changed (session: *F*(20,320) = 2.31, *P* < 0.01; session × strain: *F*(20,320) = 3.10, *P* < 0.001), and post hoc analysis indicated a significant higher level of inactive lever responding in BXD16 compared to C57BL/6J mice from the second extinction session onwards (Figure 4E). Taken together, there was no difference in extinction rate of previously active lever responding, but BXD16 mice were more active in terms of both inactive and active lever responding than C57BL/6J mice.

##### Cue-induced reinstatement of alcohol seeking behavior

Next, reinstatement of alcohol seeking by alcohol-conditioned cues was tested after extinction. In comparison with the last extinction session, the number of responses on the previously active lever increased significantly (session: *F*(1,16) = 25.18, *P* < 0.001) upon presentation of alcohol-conditioned cues during the reinstatement session (C57BL/6J: from 52.9 ± 9.5 to 85.8 ± 13.4 responses; BXD16: from 106.7 ± 17.0 to 207.2 ± 37.1 responses). This effect was strain dependent (Figure 4B; session × strain: *F*(1,16) = 6.47, *P* < 0.05). Post hoc testing indicated a significant increase in active lever responding in BXD16 (*P* = 0.003) and C57BL/6J mice (*P* = 0.020). During the reinstatement session, the preference for the previously active lever over the inactive lever increased in both strains (Figure 4D; session: *F*(1,16) = 4.64, *P* < 0.05; session × strain: *F*(1,16) = 0.47, ns). The number of previously inactive lever responses also increased (session: *F*(1,16) = 9.30, *P* < 0.01), but in contrast to active lever responses, no significant session × strain interaction was detected (session × strain: *F*(1,16) = 0.34, ns; Figure 4F). In conclusion, the high impulsive BXD16 strain showed a stronger reinstatement of alcohol seeking than the C57BL/6J strain.

## Discussion

Here we report that in the mouse BXD16 strain, poor executive control coincides with enhanced motivation for alcohol intake and proneness to cue-induced reinstatement compared with C57BL/6J mice.

Previous studies indicated that BXD16 has reduced inhibitory control in a reversal learning task (Laughlin et al., 2011) and poor attentional performance in a 5CSRTT (Loos et al., 2012). Here, we confirmed these phenotypes using a SCRTT testing protocol. BXD16 mice made more impulsive, premature Go-responses and showed increased intra-individual variability in Go-response latencies, which is an index of brief lapses in attention during which the onset of a Go-stimulus is not detected (Leth-Steensen et al., 2000), than C57BL/6J mice. Together, these data convincingly and reproducibly show reduced executive control in BXD16 mice compared with C57BL/6J mice, in the absence of preexisting differences in activity.

After the sucrose fading training procedure, BXD16 mice showed a higher intake of 10% alcohol solution compared with C57BL/6J mice. In addition, compared with C57BL/6J mice, the BXD16 strain reached higher breakpoints in the absence of differences in the preference for the active lever and number of inactive lever responses during a PR2 schedule of reinforcement. Thus, FR1 and PR2 data indicate a stronger motivation to self-administer 10% alcohol reward of BXD16 compared with C57BL/6J mice.

Under extinction conditions, responding on and preference for the previously active lever decreased in both strains at a similar rate. This indicated that persistent active lever responding for alcohol during the preceding sucrose-to-alcohol-fading procedure of BXD16 mice was not due to a failure to extinguish responding for sucrose.

During the cue-induced reinstatement session, upon exposure to a priming drop of alcohol and continuous alcohol-associated stimuli, BXD16 mice showed a more robust reinstatement of alcohol seeking than C57BL/6J mice. Thus, BXD16 mice appear more susceptible to cue-induced reinstatement, a process that seriously hampers successful treatment of alcohol addiction.

The impulsive BXD 16 strain showed enhanced TO responding compared with C57BL/6J mice. This is in line with enhanced TO responding of rats, characterized as impulsive in a 5CSRTT (Diergaarde et al., 2009), in a sucrose self-administration paradigm. In addition, enhanced TO responding has previously been observed in addiction-prone rats, both in cocaine (Deroche-Gamonet et al., 2004) and palatable food seeking paradigms (Ghitza et al., 2006) and has been interpreted as a compulsive component of reward seeking. Thus, the lack of executive control in BXD16 extends beyond dedicated attention/impulsivity tasks.

From earlier studies in mice (Loos et al., 2009) and rats (Molander et al., 2011) it is established that high levels of impulsive responding in a 5CSRT task are not related to levels of locomotor activity. Furthermore, both our own data and that of other labs^1^ indicated similar, if anything reduced activity in BXD16 compared with C57BL/6J mice (e.g., ID 10910; Yang et al., 2008; ID 10037; Bolivar and Flaherty, 2003). Observations of reduced activity in BXD16 compared with C57BL/6J may be the result of elevated levels of anxiety in novel test apparatuses, as shown by the decreased time on the open arm of the EPM and time in the brightly lit compartment of the DLB. Nonetheless, decreased or equal general activity levels in BXD16 compared with C57BL/6J mice cannot explain the increase in responding in the alcohol self-administration protocol. Nonetheless, BXD16 mice maintain higher active lever pressing for alcohol, have higher breakpoints, and have higher levels of active and inactive lever pressing than C57 mice throughout extinction, which all might be explained by differences in general activity. However, the significantly stronger increase of exclusively active lever responding in BXD16 mice during the reinstatement session compared with C57BL/6J mice clearly showed the specificity of the BXD16 activity. Thus the behavior during the reinstatement session was pivotal in establishing higher alcohol-seeking behavior in BXD16 mice compared with C57BL/6J mice.

In the current protocol the white cue light switched on upon delivery of reward, starting from the first session onwards, initially pairing this cue light with sucrose reward. The association with sucrose was however disrupted for 17 sessions (S32–S48) when reward consisted of 10% alcohol without sucrose, effectively pairing this cue light with 10% alcohol reward. In addition, during the reinstatement procedure, the white cue light was presented together with a drop of alcohol. This, so-called compound cue, will particularly re-establish the association between the cue and the alcohol reward. Nevertheless, we cannot completely rule out that the reinstatement procedure partly triggered a remote sucrose memory.

Our results, linking poor executive control to enhanced motivation and reinstatement of alcohol seeking extend previous studies in rodents, indicating that impulsive action measured in Go-NoGo tasks (Logue et al., 1998; Gubner et al., 2010) and impulsive choice in a delayed reward paradigm (Poulos et al., 1995; Wilhelm and Mitchell, 2008; Oberlin and Grahame, 2009) coincide with the consumption, preference and sensitizing effects of alcohol. However, levels of impulsive choice in rats did not predict differences in alcohol demand elasticity, showing no relation between this variety of impulsivity and the motivation to work for alcohol at increasing response requirements (Diergaarde et al., 2012), clearly indicating the complex interplay between varieties of executive functioning and specific aspects of addiction-related behavior.

The oral consumption of alcohol is accompanied by chemosensory perception of its flavor, which plays an important role in its acceptance and rejection. Like humans, rodents depend on the possibility to detect the sweet (sucrose-like) and bitter (quinine-like) taste of alcohol. BXD16 mice showed the least voluntary consumption of a bitter quinine solution of a panel of BXD strain including the parental lines (Phillips et al., 1991), indicating a good perception of bitterness, as well as a low-moderate voluntary consumption of saccharine (Lush, 1989). This indicates that enhanced alcohol self-administration cannot be explained by a reduced perception of bitterness or increased perception of sweetness of alcohol in the BXD16 strain.

Moreover, C57BL/6J mice are known to show voluntary alcohol intake and alcohol metabolism similar to, or even higher than BXD16 (Crabbe et al., 1983; Phillips et al., 1994; Rodriguez et al., 1994; Grisel et al., 2002). The online repository of BXD strains^2^indicates similar gene expression levels of alcohol dehydrogenases in the liver (Gatti et al., 2010), and similar alcohol metabolism as measured in blood (Grisel et al., 2002). Enhanced alcohol seeking in BXD16 mice in the current study may be explained by the observation that a sucrose fading protocol may overcome an initial aversive effect of alcohol in low-alcohol drinking rodents, and enhance subsequent voluntary alcohol intake (Tolliver et al., 1988). Thus, in comparison with C57BL/6J, high impulsive BXD16 mice may have an equal or lower propensity to initiate alcohol self-administration, but once alcohol self-administration is established, they show an increased motivation for alcohol and enhanced cue-induced alcohol seeking, even after a prolonged period of extinction training.

In conclusion, our study in mice links poor executive control to two prominent features of alcohol use disorder, i.e., enhanced motivation to self-administer alcohol and exaggerated reinstatement of alcohol seeking. Additional studies are required to dissect the genetic loci contributing to impulsivity and alcohol seeking, as the association of impulsivity/inattention and alcohol seeking may depend on separate genetic loci.

## Author contributions

Maarten Loos, August B. Smit, Taco J. De Vries and Sabine Spijker designed the experiments and wrote the paper. Maarten Loos and Jorn Staal performed the experiments and analyzed the data.

## Conflict of interest statement

The authors declare that the research was conducted in the absence of any commercial or financial relationships that could be construed as a potential conflict of interest.

## Footnotes

^1^WebQTL; www.genenetwork.org

^2^WebQTL; www.genenetwork.org
